# A retrospective cohort study on the use of machine learning to predict stone-free status following percutaneous nephrolithotomy: An experience from Saudi Arabia

**DOI:** 10.1016/j.amsu.2022.104957

**Published:** 2022-11-17

**Authors:** Mohammad A. Alghafees, Saleha Abdul Rab, Abdulaziz S. Aljurayyad, Tariq S. Alotaibi, Belal Nedal Sabbah, Raouf M. Seyam, Lama H. Aldosari, Mohammad A. Alomar

**Affiliations:** a College of Medicine, King Saud Bin Abdulaziz University for Health Sciences, Riyadh, Saudi Arabia; b College of Medicine, Alfaisal University, Riyadh, Saudi Arabia; c Department of Urology, King Saud University Medical City, Riyadh, Saudi Arabia; d Department of Urology, King Faisal Specialist Hospital and Research Centre, Riyadh, Saudi Arabia; e Department of Urology, King Fahad University Hospital, Al-Khobar, Saudi Arabia

**Keywords:** Artificial intelligence, Machine learning, Renal stone, Stone-free status prediction, Percutaneous nephrolithotomy, Urolithiasis

## Abstract

**Background:**

Machine learning techniques have been used extensively in the field of clinical medicine, especially when used for the construction of prediction models. The aim of the study was to use machine learning to predict the stone-free status after percutaneous nephrolithotomy (PCNL).

**Materials and methods:**

This is a retrospective cohort study of 137 patients. Data from adult patients who underwent PCNL at our institute were used for the purpose of this study. Three supervised machine learning algorithms were employed: Logistic Regression, XGBoost Regressor, and Random Forests. A set of variables comprising independent attributes including age, gender, body mass index (BMI), chronic kidney disease (CKD), hypertension (HTN), diabetes mellitus, gout, renal and stone factors (previous surgery, stone location, size, and staghorn status), and pre-operative surgical factors (infections, stent, hemoglobin, creatinine, and bacteriuria) were entered.

**Results:**

137 patients were identified. The majority were males (65.4%; n = 89), aged 50 years and above (41.9%; n = 57). The stone-free status (SFS) rate was 86% (n = 118). An inverse relation was detected between SFS, and CKD and HTN. The accuracies were 71.4%, 74.5% and 75% using Logistic Regression, XGBoost, and Random Forest algorithms, respectively. Stone size, pre-operative hemoglobin, pre-operative creatinine, and stone type were the most important factors in predicting the SFS following PCNL.

**Conclusion:**

The Random Forest model showed the highest efficacy in predicting SFS. We developed an effective machine learning model to assist physicians and other healthcare professionals in selecting patients with renal stones who are most likely to have successful PCNL treatment based on their demographics and stone characteristics. Larger multicenter studies are needed to develop more powerful algorithms, such as deep learning and other AI subsets.

## Highlights

• Urolithiasis is an exceedingly common condition and requires prompt treatment and counseling to prevent recurrence.
• Artificial intelligence has great utility in predicting surgical outcomes and improving patient care in urolithiasis.
• Machine learning-based algorithms represent promising tools for the prediction of stone-free success rates after PCNL.
• The Random Forest machine learning model exhibits the highest efficacy and accuracy in predicting stone-free status.

## 1 Introduction

Nephrolithiasis, or kidney stone formation, refers to the impaction of urinary calculi along any part of the urogenital tract. It is one of the most common urogenital diseases and has seen a drastic increase in global prevalence in the last several years [[1], [2], [3]]. Particularly in Saudi Arabia, the prevalence stands at 9.1% [4], posing a considerable national burden. Management of kidney stones greatly depends on their type, shape, size, and location. While smaller renal stones (≤10 mm) are managed conservatively via symptomatic treatment, larger stones (>10 mm) require interventional management [5]. In this era of minimally invasive surgery, percutaneous nephrolithotomy (PCNL) is the treatment of choice for such stones.

PCNL refers to the insertion of a nephroscope percutaneously into the renal pelvis under ultrasonographic and/or fluoroscopic guidance to remove stones, while causing the least harm to the patient. However, several factors can impact PCNL treatment and stone-free outcome, including a history of ipsilateral open renal stone surgery, stone burden, size of the stone, localization of the stone, and number of previous percutaneous interventions [6,7]. Due to the increasing necessity of PCNL, several authors have developed scoring systems and machine learning (ML) based systems to allow prediction of stone-free status (SFS) following PCNL, thereby facilitating clinical decision-making and patient counseling [[8], [9], [10], [11]]. ML in particular utilizes artificial intelligence (AI) to “learn from” and make accurate predictions using various data sources [12], allowing clinicians to identify at-risk patients and forecast their complications in order to minimize their health risks. This makes ML algorithms helpful in the diagnosis and outcome prediction of PCNL. As a result, greater emphasis is being placed on the prediction of prospective kidney stones, or in our case, failure of PCNL therapy, as it enables early prevention and/or cure of the stone.

In this study, we aimed to validate the output of ML-based algorithms as intelligible interfaces capable of predicting stone-free post-PCNL outcomes, identify the algorithm that was most effective at doing so, and determine the most important factors in predicting SFS. Three types of supervised machine learning algorithms were employed in this study, including Logistic Regression, XGBoost Regressor, and Random Forests (RF). A set of input variables comprising independent attributes including age, gender, body mass index (BMI), chronic kidney disease (CKD), hypertension, diabetes mellitus, gout, renal and stone factors (previous surgery, stone location, size, and staghorn status), and pre-operative surgical factors (infections, stent, hemoglobin, creatinine, and bacteriuria) were entered. We then compared the performance and accuracy of all three algorithms in determining SFS. To our knowledge, this is the first comparative analysis of three AI systems used to predict stone-free outcome post-PCNL with identification of the most important predictors. This study is reported in compliance with the STROCSS criteria [13].

## 2 Materials and methods

### 2.1 Dataset

Data for all adult patients who underwent PCNL at King Faisal Specialist Hospital, Riyadh, Saudi Arabia, performed by the same consultant between the years 2020–2022, were enrolled in this study. All patients were followed for 12 months, to confirm stone clearance, alleviation of symptoms, and proper hydration and diet to prevent recurrence. Their data was extracted and surveyed to determine the preoperative and postoperative variables, and the SFS was assessed shortly after the procedure. The King Faisal Specialist Hospital and Research Centre waived IRB approval for this retrospective cohort study. This study has been registered on the Research Registry under the UIN **researchregistry8453** [14]. The results of the SFS were entered as binary numbers: 1 (stone residual i.e. Yes) and 0 (clinically insignificant residual stone fragments). The machine learning models were fitted using scikit-learn 0.18 modules of python throughout this study. The original data set is randomly divided into the 80% of the training set, and the 20% of the test set at 8:2 (108: 28).

The RF model is a decision tree-based machine learning model. Each node of the decision tree divides the data into two groups by using a cutoff value inside one of the features. By building an ensemble of randomized decision trees, each of which overfits the data and averages the results to obtain a better classification, the RF technique can reduce the effect of the overfitting problem.

### 2.2 Data analysis

The patient's data were analyzed using RF, which is one of the most used supervised machine learning method due to its simplicity, flexibility, and namely, its accuracy. It is also highly in use because of its ability to combine both a classification and regression tasks [15]. Extreme gradient boosting trees (XGBoost) was also used, which is built based on decision tree–based gradient boosting regression method. In this case, trees for prediction are built sequentially such that each subsequent tree aims to reduce the errors from their predecessors [16] and light gradient boosting method (LightGBM). This is similar to the LightGBM model as it is an ensemble technique which combines weak individual models to form a single accurate model [17]. The machine learning models were trained on a dataset with a binary classification output predicting the target SFS. In the initial dataset, the stone-free column was a multi-class output (Yes, Insignificant fragment, No). Afterwards, the Pandas mapping function was employed to combine both “Insignificant fragment” and “No” together into a single output, thereby making the taking column a complete binary classification output column. 80% of the dataset was used in training of the model and 20% of the model was used in test (validation) of the model. For this experiment, the learning rate for the models was tuned at 0.001 and GridSearchCV was employed in selecting the best parameters for the models to be trained on. This strategy enabled the predictive model to be able to learn using the best features and parameters from the dataset, build individual weak models from them and finally generate a stronger model as a combination of the several weak models. To calculate the Are Under Curve, sklearn.metric module in Python was used. In addition, a multivariate analysis was performed using Matplotlib, a built-in library in Python, used for data visualization. This enabled us to understand how the comorbidities impacted the status of the kidney stone after the PCNL treatment.

## 3 Results

137 patients were identified. The mean age of the patients who participated in the study was 44.8. More than half (65.4%) of the patients were male. Majority (97.1%) of the patients had CKD, about two-thirds (77.2%) of the patients had hypertension, and similarly 71.3% had diabetes mellitus. The number of stone-free cases after PCNL was 118 (86%). Table 1 shows the full demographic characteristics.

**Table 1:** Socio-demographic characteristics and co-morbidities of patients.

Variables		Frequency	Percentage (%)
Age	Under 20	12	9.6
	21–30 years	16	11.8
	31–40 years	17	12.5
	41–50 years	33	24.2
	50 and above	57	41.9
Gender	Male	89	65.4
	Female	47	34.6
Chronic Kidney Disease	No	132	97.1
	Yes	4	2.9
Hypertension	No	105	77.2
	Yes	31	22.8
Diabetes Mellitus	No	97	71.3
	Yes	39	28.7

**Fig. 1:**
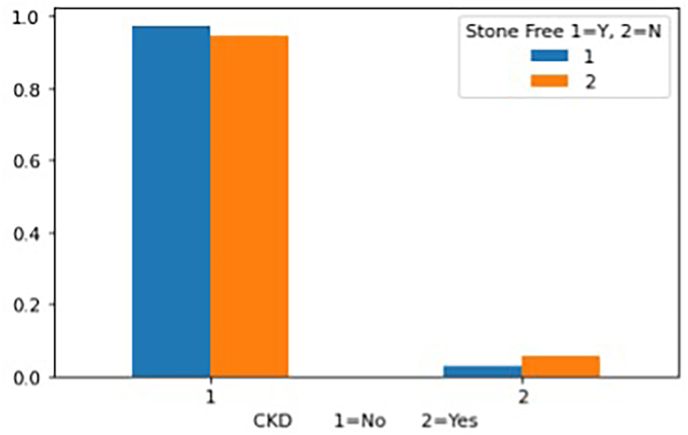
A chart showing the relationship between CKD and stone-free status.

**Fig. 2:**
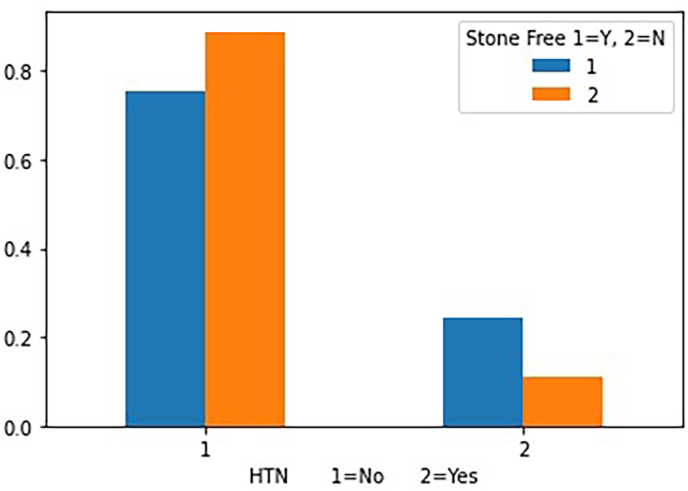
The relationship between hypertension and stone-free status.

Fig. 1 describes the relationship between CKD and SFS, revealing that the majority of patients who became stone-free after PCNL were patients who did not have CKD listed as a comorbidity. Fig. 2 reveals the state of patients with hypertension (HTN) who became stone-free after the PCNL treatment. It shows that a large percentage of patients with hypertension were not stone-free after the PCNL treatment.

### 3.1 Stone characteristics

Our results revealed the different characteristics of the stones present in our patients undergoing PCNL treatment was conducted and was used for prediction in the machine learning model. This showed that the average stone size (burden) of each stone retrieved measured lengthwise was 38.8 ± 15.46 mm and widthwise was 14.4 ± 7.54 mm. The table also revealed that the average number of stone in each patient reviewed for the PCNL treatment is 3.

### 3.2 Performance evaluation

**Fig. 3:**
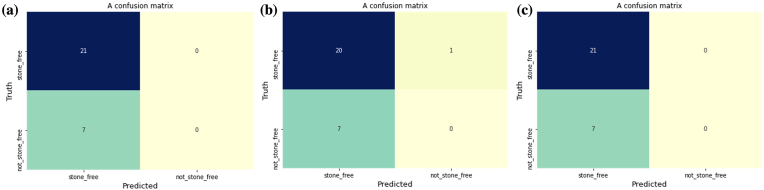
This figure shows the confusion matrix for each of the models, describing the specificity of the model in terms of how true the predicted values were accurate to their original values. Fig. 3 (a), which represents Logistic Regression, shows that the model predicts 20 patients to be stone-free and in the real sense they were stone-free, however, it predicts 1 patient to still have a stone, but in the real sense the patient did not have the stone. In contrast **(b)**, which represents XGBoost, and **(c)**, which represents Random Forest, both adequately predict all patients that are stone-free to be stone-free. Hence, the specificity of both models is higher than that of the logistic regression.

The prediction accuracies of the models used to predict stone-free success were 71.4%, 74.5%, and 75% using Logistic Regression, XGBoost, and Random Forest (RF) algorithms respectively. In predictions for the target label, the stone-free Random Forest performed better due to their higher accuracy. This is due to the sophisticated nature of the algorithm and its ability to learn from individual weaker models to form a stronger one. The evaluation of the models using Area Under Curve (AUC) for the RF offered higher value than XGBoost and logistic regression. Fig. 3a–c shows the confusion matrix for each of the models which explains the specificity of the model in terms of how true the predicted values are accurate to the original values. In Fig. 3a, it shows that the model predicts 20 patients to be stone-free and in the real sense they were stone-free, however, it predicts 1 patient to still have a stone but in the real sense the patient did not have the stone. In contrast, Fig. 3b, which represents XGBoost, and Fig. 3c, which represents Random Forest, both adequately predict all patients that are stone-free to be stone-free. Hence, the specificity of both models is higher than that of the logistic regression.

## 4 Discussion

Nephrolithiasis poses an exceedingly significant burden on individuals and society, making its precise diagnosis and treatment a necessity. There are several risk factors for kidney stone formation, which also influence the type and size of stone formed, including gender, body mass index (BMI), diabetes mellitus, gout, hypertension, a history of nephrolithiasis, prior abdominal surgery, and others [18,19]. Particularly for larger, more painful stones, surgical intervention is often required, including extracorporeal shock wave lithotripsy (ESWL), ureterorenoscopy, and PCNL – of which PCNL has been established to be the least invasive, and is considered standard care for largest stones [20,21]. However, the success rate of PCNL in producing satisfactory outcomes (i.e., SFS) without stone recurrence or requiring stent placement is dependent on several factors, such as characteristics of the stone (size, location, staghorn status, type of stone), surgical history, and patient characteristics. Thereby, prediction of post-PCNL outcome can be highly useful, paving the way for improved patient counseling, patient selection, and postoperative care [11].

Recently, several authors have developed scoring systems that assist in predicting post-PCNL stone-free rates, such as the qualitative Guy's stone score (GSS) as introduced by Thomas et al. [22], the more quantitative Clinical Research Office of the Endourological Society (CROES) nomogram established by Smith and colleagues [23], and the STONE scoring system proposed by Okhunov et al. [24]. While these scoring systems have proven to be quite effective, a systematic review by Withington et al. revealed that the performance of all systems in predicting SFS was similar, and their ability to predict post-PCNL adverse effects was questionable, highlighting the need for further improvement in predicting systems [25]. Recent data have revealed that the use of machine learning-based algorithms to predict stone-free outcome has shown promising results and may replace scoring systems altogether.

In a study by Zhao et al. six stone characteristics (stone burden, number, location, multiple, staghorn form and institute-level case volume) were employed to design a prediction model, which was then compared to the GSS and STONE scoring system. It was established that the prediction accuracy of ML models was superior to that of the STONE scoring system, and that ML models overall had AUC not inferior to GSS or STONE scoring systems [26]. Additionally, in a study by Aminsharifi et al. a complex nonlinear mathematical model was trained to predict stone-free status, with the accuracy and sensitivity of the system ranging from 81% to 98.2% (a more favorable outcome than our own of 71.4–75%). However, it is worth noting that this study's sample size was higher than ours (254 vs. 137), while the SFS was lower (76.4% vs. 86%) [27]. A second study conducted by Aminsharifi et al. produced an ML model with higher prediction accuracy than that of the GSS or STONE scoring system [11]. This study utilized the support vector machine model, and revealed that the AUC for the machine learning software (0.915) was significantly larger than the AUC for GSS (0.615) or CROES (0.621) nomograms (p < 0.001). The ML algorithm determined stone burden, the presence of staghorn calculi, or multiple renal stones as the most highly weighted preoperative factors affecting the post-PCNL SFS, supporting our result of stone size being one of the most important post-PCNL SFS determinants.

Furthermore, some authors have successfully developed ML-based decision support systems (DSS) for the prediction of the postoperative outcomes of PCNL [28,29]. It has been established that DSS can serve as a useful tool to give clinicians and surgeons more insight into the patient's condition and allow them to provide counseling preoperatively, predict post-PCNL outcomes, and ultimately choose the appropriate surgical management for the patient. Additionally, the DSS accurately predicted the outcomes of the PCNL with favorable accuracy (81%) [29], as well as procedure postoperative ancillary procedures with accuracies ranging from 85.2% to 95.2% [28], further highlighting the largely unexplored potential utility of ML-based systems.

With regards to analyzing our dataset, three ML models were utilized including RF, XGBoost, and Logistic Regression. While no similar comparative analysis exists on ML models used to predict SFS post-PNCL, for the sake of illustration it is worth comparing our results to a study conducted by Yang et al. who also compared the accuracy of three ML models, namely RF, XGBoost, and LightGBM, in predicting stone-free success in patients undergoing ESWL [30]. As established by this study, LightGBM had the highest prediction accuracy. This contrasts with our finding of RF having the highest accuracy, although we believe this discrepancy is owed to the difference in procedure performed, as well as the variables used. However, this does highlight that further research is needed to determine the most accurate ML algorithm for predicting SFS outcomes for all three surgical interventions: ESWL, ureterorenoscopy, and PCNL.

Lastly, the foundation of ML is a statistical structure. Various methods are used to achieve the best accurate prediction possible. Our study reveals that ML is effective in predicting the output of patients with kidney stones following a PCNL procedure. Although the AUC of our models were between the range of 0.5 and 0.69, the accuracies were on the high end, indicating that the models have worthy capabilities of prediction. Random Forests, ensemble approaches, decision trees, artificial neural networks, computer vision, and deep learning are just a few of the algorithms used in ML. In this work, we used three algorithms to estimate stone-free status, and all three performed admirably, establishing that ML algorithms are an effective tool for predicting stone-free rates following PCNL using AUCs.

### 4.1 Factors contributing to stone-free status

Another benefit of ML models is their ability to identify exact parameters that are most crucial to making the correct diagnosis and postoperative outcome, based on their robust learning ability and understanding of the dataset. The RF model was determined to have the best prediction accuracy. It is evident that size, pre-operative hemoglobin, pre-operative creatinine, and stone type were the most important factors in predicting SFS following PCNL. This is then followed by features like the number of stones and a prior treatment of the same stone. Taken altogether, this data is of great benefit, and can potentially help avoid wastage of resources, enable quick and easy diagnoses, lower the cost of unnecessary tests and scans, and ultimately simplify healthcare delivery. Thereby our data carries great utility in facilitating clinical decisions and should be compared to other statistical methods.

Additionally, consistent with our results, several other studies also found stone type and stone size to be statistically significant factors affecting SFS [31,32]. For example, Atmoko et al. determined that stone burden (size >52 mm) and a history of treatment for the same stone were significant predictors, similar to our findings [6]. Another study by El-Nahas et al. that included only patients with staghorn stones found that stone characteristics such as complete staghorn and secondary calyceal stones were independent risk factors for residual stones after PCNL [33].

Generally, while considering kidney stones as well as PCNL in the field of AI, the number of studies describing machine learning methods to predict operative outcomes or assist with operative decisions are limited. Therefore, it is imperative to improve existing ML methods, as well as investigate further what new models may bring to the table. In order to improve the efficacy of ML models, two things must be established: (1) that more domain-knowledge professionals, including urologists, statisticians, and computer scientists, must be involved in this field of endourology, and (2) that patients should be implored to participate in such studies. Furthermore, greater data from various regions or demographics should be collected in order to forecast future events. To our knowledge, the presented study in the first of its kind in the Gulf region, known for its high stone burden [34]. There is a need to establish, administer, and share a cross-country or national endourology database, as it will help with diagnosis and prediction utilizing AI systems. This study would add to existing knowledge on kidney stone prediction using ML methods. It might also serve as baseline data for intervention assessment by other researchers in other facilities.

### 4.2 Limitations

The generalizability of classifiers was tested using a data set of roughly 28 patients who had preoperative and postoperative follow-ups at the same centre and whose PCNL surgery success and failure were established using a computed tomography scan. The testing data set was not included in the model's training data set. To increase the learning rate and depth of recursion on the dataset, we used hyperparameter tuning for the models.

## 5 Conclusion

We developed an effective ML model to assist physicians and other healthcare professionals in selecting patients with renal stones who are most likely to have successful PCNL treatment based on their demographics and stone characteristics and have shown that the prediction accuracy can be as high as 75% or higher if proper and adequate data is collected, particularly on the stone characteristics and the patient's co-morbidities. The suggested ML model can help clinicians and decision-makers plan for PCNL treatment. Supplemented with more powerful and newer algorithms, such as deep learning and other AI subsets, these ML models will improve the prediction rate dramatically. This will translate into early diagnosis of stone for patients, improved prognosis, and establishment of novel methods of treating and preventing similar occurrence.

## Ethical approval

The King Faisal Specialist Hospital and Research Centre (KFSH&RC) waived IRB approval for this study.

## Sources of funding

No funding was provided.

## Author contribution

M.A.Algh, M.A.Alo contributed to study concept and design, data collection and analysis. A.S.A, T.S.A, S.A.R, B.N.S drafted the manuscript. S.A.R, B.N.S, R.M.S, L.H.A contributed to reviewing and finalizing the manuscript. All authors reviewed the manuscript for intellectual content and approved the submission.

## Conflicts of interest

The authors declare no conflicts of interest.

## Registration of research studies

Name of the registry: Research Registry.
Unique Identifying number or registration ID: researchregistry8453.
Hyperlink to your specific registration (must be publicly accessible and will be checked): https://www.researchregistry.com/browse-the-registry#home/registrationdetails/63623d9251f2db002183a03e/

## Guarantor

Mohammad A. Alghafees.

## Consent

Not applicable.

## Provenance and peer review

Not commissioned, externally peer-reviewed.
